# Sulfate-Containing Composite Based on Ni-Rich Layered Oxide LiNi_0.8_Mn_0.1_Co_0.1_O_2_ as High-Performance Cathode Material for Li-ion Batteries

**DOI:** 10.3390/nano10122381

**Published:** 2020-11-29

**Authors:** Aleksandra A. Savina, Elena D. Orlova, Anatolii V. Morozov, Sergey Yu. Luchkin, Artem M. Abakumov

**Affiliations:** Center for Energy Science and Technology, Skolkovo Institute of Science and Technology, Bolshoy Boulevard 30, bld. 1, 121205 Moscow, Russia; A.Savina@skoltech.ru (A.A.S.); Elena.Orlova2@skoltech.ru (E.D.O.); Anatolii.Morozov@skoltech.ru (A.V.M.); s.luchkin@skoltech.ru (S.Y.L.)

**Keywords:** cathode, Li-ion battery, layered oxide, Ni-rich NMC, sulfur-containing composite, Li_2_SO_4_, grain boundary

## Abstract

Composite positive electrode materials (1−*x*) LiNi_0.8_Mn_0.1_Co_0.1_O_2_∙*x*Li_2_SO_4_ (*x* = 0.002–0.005) for Li-ion batteries have been synthesized via conventional hydroxide or carbonate coprecipitation routes with subsequent high-temperature lithiation in either air or oxygen atmosphere. A comparative study of the materials prepared from transition metal sulfates (i.e., containing sulfur) and acetates (i.e., sulfur-free) with powder X-ray diffraction, electron diffraction, high angle annular dark field transmission electron microscopy, energy-dispersive X-ray spectroscopy, and electron energy loss spectroscopy revealed that the sulfur-containing species occur as amorphous Li_2_SO_4_ at the grain boundaries and intergranular contacts of the primary NMC811 crystallites. This results in a noticeable enhancement of rate capability and capacity retention over prolonged charge/discharge cycling compared to their sulfur-free analogs. The improvement is attributed to suppressing the high voltage phase transition and the associated accumulation of anti-site disorder upon cycling and improving the secondary agglomerates’ mechanical integrity by increasing interfacial fracture toughness through linking primary NMC811 particles with soft Li_2_SO_4_ binder, as demonstrated with nanoindentation experiments. As the synthesis of the (1−*x*) LiNi_0.8_Mn_0.1_Co_0.1_O_2_∙*x*Li_2_SO_4_ composites do not require additional operational steps to introduce sulfur, these electrode materials might demonstrate high potential for commercialization.

## 1. Introduction

The production of battery-electric (EVs) and hybrid-electric vehicles (HEVs) has intensively grown since they provide ecologically more friendly transportation. Among currently used electrochemical energy storage devices, lithium-ion batteries (LIBs) dominate the battery market as LIBs possess high energy density, fast recharging capability, and high discharge power [1]. However, to meet the growing requirements such as charging time reduction, increased driving range, and safety, further improvements of LIBs towards increased energy density, rate capability, and cycle stability are needed [2]. Amid the three main components of the LIBs (cathode, anode, and electrolyte), the positive electrode (cathode) material is known to be a limiting factor in both performance and cost of the battery. Among many cathode materials, the layered oxides of nickel, manganese, and cobalt LiNi*_x_*Co*_y_*Mn*_z_*O_2_ with high Ni content (*x* ≥ 0.6), termed Ni-rich NMCs, are considered to be promising candidates for high-energy cathode materials for the next generation of LIBs because of their high discharge capacity and low cost. Notably, such cathode materials demonstrate relatively high structural and chemical stability at elevated operation voltage (4.3 V vs. Li/Li^+^) compared to the commercialized LiCoO_2_, enabling extraction of more Li ions from their structure. For instance, typical Ni-rich layered oxide LiNi_0.8_Mn_0.1_Co_0.1_O_2_ (NMC811) delivers a high practical discharge capacity of ~200 mA∙g^−1^ at 4.3 V vs. Li/Li^+^ [3]. However, the practical application of Ni-rich NMCs is impeded mainly by significant drawbacks, such as high initial irreversible capacity loss, short cycle life, poor thermal stability, and safety issues, which may appear during the whole battery life [3,4]. Undesirable changes in the crystal structure and chemical composition of the Ni-rich NMC cathodes during charge/discharge cycling are believed to be the main reason for these disadvantages [5]. The structural instability of Ni-rich NMCs during the charge/discharge process associates with transition metals (TM) cation migration into lithium layers leading to the gradual transformation of the layered structure into a spinel-like structure, and then, finally, into an electrochemically inactive product with the rock-salt type disordered structure. The partially disordered phase has smaller interlayer spacing and a higher activation energy barrier for Li^+^ migration than the well-ordered layered phase. This leads to a drop of Li diffusivity that, in turn, decreases the electrochemical performance of the cathode material [6]. Moreover, structural disordering is accompanied by oxygen release that may trigger severe safety issues, such as thermal runaway, fire, and explosions [5,7,8]. The mentioned phase transitions with TM ions migration are accompanied by changes in the unit cell volume during charge and discharge, causing microcracks within the primary NMC particles. As the primary particles are merged into secondary agglomerates, cracks also propagate along the grain boundaries [9]. Mechanical destruction contributes to rapid capacity fading, ascribed to the deterioration of the ionic and electronic conduction network [10].

If the bulk structure changes are shown to be reversible [11], the surface of the Ni-rich NMCs particles undergoes significant irreversible structural rearrangements caused by undesired side reactions at the interface between the electrode and electrolyte. First, electrochemically inactive lithium residues, formed at the surface after solid-state synthesis of the layered oxides, can act like an ion/electron blocker [12]. Second, hydrolysis of the electrolyte salt LiPF_6_ by a trace amount of moisture can cause HF’s formation, leading to the dissolution of TM ions from the cathode material surface into the electrolyte. In addition, an ever-increasing amount of dissolved TM ions, depositing on the anode material, hinders the migration of lithium ions and also contributes to the accumulation of electrolytic decomposition products on the negative electrode [13]. Thus, these side reactions profoundly impact the lithiation kinetics at the electrode/electrolyte interface and trigger a substantial decline in capacity retention.

In order to resolve the mentioned problems, a lot of surface treatment and morphology control technologies have been suggested. The introduction of additional layers consisting of metal oxides [14], fluorides [15], or polymers [16] on the surface of the secondary particles is the most common approach. However, these coating materials are usually poor Li-ion conductors. Although good cyclability is achieved, the rate capability, which mainly depends on the electrode materials’ ionic conductivity, is usually insufficient. One of the approaches applied in all-solid-state batteries aimed at solving the conductivity problem is adding at least 20 mol% of amorphous matrixes into cathode materials [17]. Despite such modification increasing both electronic and ionic conductivities, this method is expected to decline energy density. The fundamental issues related to the mechanical degradation of cathode materials during cycling are also not completely resolved by the mentioned approaches. Development of full concentration-gradient phases [18] and the grain boundary coating, including the nanoscale surface treatment of primary particles [19], lead to the improved structural and thermal stability of Ni-rich NMCs. However, these methods require complex synthesis and/or advanced equipment, which cause an increase of process time and cost.

In the present work, a highly stable composite cathode material (1−*x*) LiNi_0.8_Mn_0.1_Co_0.1_O_2_∙*x*Li_2_SO_4_, obtained via the coprecipitation method without any special surface coating procedure, is reported. Integration of amorphous Li_2_SO_4_ into Ni-rich NMC secondary particles, predominantly to the boundaries between the primary particles and their intergranular contacts, increases the secondary agglomerates’ interfacial fracture toughness. Thus, it suppresses mechanical fracture of the cathode particles during electrochemical cycling. The composite cathode possesses enhanced capacity retention and rate capability compared to single-phase materials due to surface protection from reaction with the electrolyte and suppressing anti-site disorder. Simultaneously, the composite cathode material synthesis does not include additional time- and cost-consuming operational steps, which makes such material attractive for industry needs.

## 2. Materials and Methods

The layered LiNi_0.8_Mn_0.1_Co_0.1_O_2_ oxide materials were synthesized via a coprecipitation method followed by high-temperature lithiation in either air or oxygen atmosphere. First, hydroxide or carbonate precursors were precipitated from aqueous solutions of either TM sulfates (NiSO_4_·6H_2_O, MnSO_4_·H_2_O and CoSO_4_·7H_2_O, Sigma Aldrich, (St. Louis, MO, USA), ≥99%) or acetates ((CH_3_COO)_2_Ni·4H_2_O, (CH_3_COO)_2_Mn·4H_2_O and (CH_3_COO)_2_Co·4H_2_O, Sigma Aldrich, (St. Louis, MO, USA), ≥99%). Aqueous solutions of either TM sulfates or acetates (the molar ratio of Ni:Mn:Co = 8:1:1) with total concentrations of TMs given in Table 1 were pumped slowly into a continuously stirred batch reactor under an Ar atmosphere. Simultaneously, an aqueous solution of either NaOH or Na_2_CO_3_ and NH_3_·H_2_O with the concentrations provided in Table 1 was pumped into the reactor as precipitating and chelating agents, respectively. All solutions were pumped separately into the reactor at a feeding rate of about 0.6 L h^−1^, and continuous stirring was kept at 800 r min^−1^. The reactor temperature and pH value temperature were fixed to 50 °C and 11.5 for the hydroxide route and 56 °C and 7.8 for the carbonate route. The precipitated precursors were filtered, carefully washed several times with deionized water, and dried at 110 °C for 12 h under a dynamic vacuum. The obtained hydroxide or carbonate precursors and LiOH·H_2_O were mixed with a molar ratio of 1:1.06 or 1:1.03, respectively, and annealed at 500 °C for 5 h and then at 850 °C for 12 h in air with intermediate grinding between the calcination steps, or at 750 °C for 12 h in flowing oxygen atmosphere. All samples were further indicated with two capital letters and a calcination atmosphere in brackets. The first letter corresponded to the type of precipitated precursor (either hydroxide (H) or carbonate (C)), the second letter corresponded to the TM source used for precipitation (either sulfates (S) or acetates (A)); the calcination atmosphere was denoted as either air (a) or oxygen (o). Therefore, eight samples in total were discussed further: HS(a), HA(a), CS(a), CA(a), HS(o), HA(o), CS(o) and CA(o).

X-ray diffraction (XRD) patterns of powder samples were obtained by a Huber G670 Guinier diffractometer (Rimsting, Germany) using Co-K_α1_ radiation (λ = 1.78892Å), a curved Ge (111) monochromator, and an image plate detector. The powder XRD data were collected at room temperature over the 10°–90° 2θ range with the step of 0.005°. The unit cell parameters were calculated using the Le Bail method. Also, crystal structures were refined using the Rietveld method. Le Bail decomposition and Rietveld analysis were performed with the JANA2006 program package (Prague, Czech Republic, Version string 25/10/2015) [20].

Particle morphology and size distribution in the pristine and cycled cathodes were examined with scanning electron microscopy (SEM) using a ThermoFisher Quattro S microscope (Waltham, MA, USA).

Electron diffraction (ED) patterns, high-angle annular dark-field scanning transmission electron microscopy (HAADF-STEM) images, compositional maps, obtained by energy-dispersive X-ray spectroscopy in a STEM mode (EDS-STEM), and electron energy loss spectra (EELS) were acquired with a ThermoFisher Titan Themis Z transmission electron microscope (Eindhoven, The Netherlands) at 200 kV. It was equipped with a Super-X system for energy-dispersive X-ray spectroscopy and a Gatan Quantum ER965 spectrometer (Pleasanton, CA, USA) for EELS. TEM samples were prepared in air by crushing the crystals with an agate mortar and pestle in ethanol and depositing drops of suspension onto a carbon film supported by a copper grid.

Atomic force microscopy (AFM) nanoindentation and imaging were performed on cross-sections of the samples using a Cypher ES AFM (Asylum Research, Oxford Instruments, Santa Barbara, CA, USA) installed in an Ar-filled glovebox. The nanoindentation was made by a single crystal diamond probe with the 4-sided pyramid tip with a 45 ± 10° half angle at the tip apex (DRP_In). The imaging was made by a single crystal diamond probe (HA_NC/FD). The cross-sections were prepared by embedding the electrodes into epoxy resin and consequent mechanical polishing.

The electrochemical properties were tested in coin-type 2032R cells using MTI BST battery analyzers (Shenzhen, China) with lithium metal as a counter electrode. The active cathode material, conductive additive (Super-P), and binder (polyvinylidene-fluoride, PVDF) were suspended in N-methyl-2-pyrrolidone (NMP) with a weight ratio of 80:10:10 and homogenized to form a slurry. The obtained slurry was applied onto a carbon-coated Al current collector (Xiamen, Fujian, China) using automatic film applicator Zehntner ZAA 2300 (Schwerzenbach, Switzerland), dried, and then cut into 16 mm diameter disks with cathode loading level of 6–7 mg cm^−2^. The electrolyte consisting of 1M LiPF_6_ (Sigma-Aldrich, St. Louis, MO, USA, ≥99.99%) solution in ethylene carbonate/propylene carbonate/dimethyl carbonate solution (EC:PC:DMC = 1:1:3 vol.) was used. The half-cells were assembled in an Ar-filled glove box, and galvanostatic tests were performed in the potential range of 2.7–4.3 V vs. Li/Li^+^ at room temperature at different current densities, under the following program: 5 cycles at 0.1C, 5 cycles at 0.2C, 5 cycles at 0.5C, 5 cycles at 0.2C, 5 cycles at 1C, 5 cycles at 0.2C, and 100 cycles at 1C, where 1C = 200 mA g^−1^.

## 3. Results

**Figure 1:**
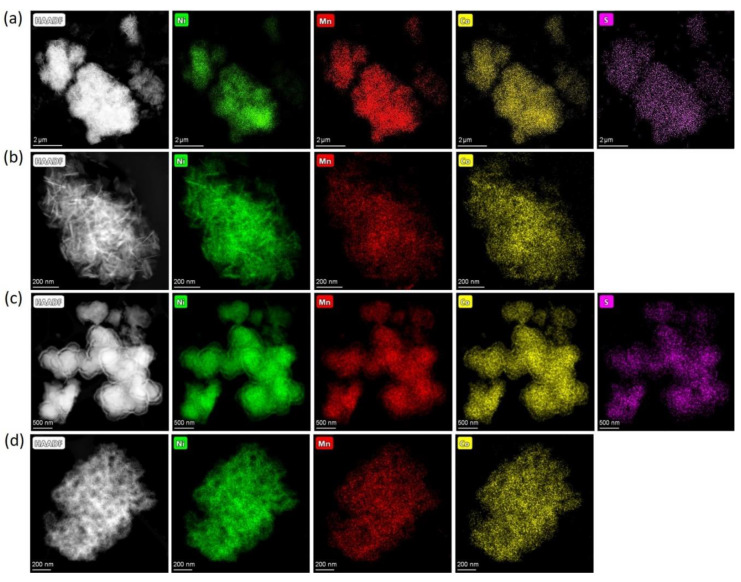
HAADF-STEM images of the (**a**) HS, (**b**) HA, (**c**) CS, (**d**) CA precursors with the compositional EDS maps of nickel, manganese, cobalt, and sulfur.

Powder XRD patterns of the obtained precursors are illustrated in Figure S1 of Supplementary Materials. Pairs of hydroxide precursors, HS and HA, and carbonate precursors, CS and CA, had identical powder XRD patterns. The hydroxide precursors precipitated from either sulfates or acetates demonstrate the β-Ni(OH)_2_-type structure (ICDD #74-2075) for which all diffraction peaks were indexed to a trigonal structure with the *P*3*m*1 space group and refined lattice parameters *a* = 3.121(1), *c* = 4.612(6) Å (Figure S1a). The absence of impurity phases indicates that the Co^2+^ and Mn^2+^ cations partially substituted Ni^2+^ in the β-Ni(OH)_2_ structure. Although the carbonate precursor’s crystallinity was lower, the strongest diffraction peaks matched the powder XRD pattern of NiCO_3_∙H_2_O (ICDD #12-0276). According to EDS-STEM compositional mapping, all precursors possessed homogeneous distribution of Ni, Mn, and Co (Figure 1) with the metal atomic ratio corresponding to the desired NMC811 stoichiometry. Additionally, EDS-STEM maps demonstrated that the precursors precipitated from sulfates contain sulfur, uniformly distributed throughout the material (Figure 1a,c). The sulfur content and Ni:Mn:Co atomic ratio for the precursors are presented in Table 2.

The effect of TM source and precursor form on the morphology of NMC811 was examined by scanning electron microscopy (SEM). Both carbonate and hydroxide precursors precipitated from sulfates and acetates consisting of secondary particles with irregular shapes with an average size of 8–12 µm (Figure S2a,c,e,g). These secondary particles were composed of numerous agglomerated flakes forming 3D hierarchical rose-like nanostructures in the hydroxide precursors (Figure S2b,d), while both carbonate precursor particles were composed of flakes and small sphere-like particles (Figure S2f,h). Although the differences in microstructural organization of secondary agglomerates in the precursors were clear in the SEM images, they were not fully inherited in the microstructure of the final NMC811 products, as will be demonstrated later.

**Figure 2:**
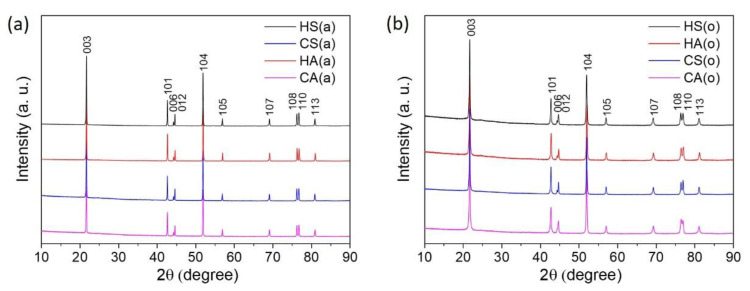
Powder XRD patterns of the NMC811 samples (**a**) HS(a), HA(a), CS(a) and CA(a); (**b**) HS(o), HA(o), CS(o), and CA(o).

In order to obtain the final NMC811 materials, the precursors and LiOH∙H_2_O were subjected to heat treatment in two different atmospheres-air and oxygen. It is generally considered that the oxygen annealing atmosphere should suppress Li and TM cation mixing (anti-site disorder) during synthesis, helping maintain a high content of Ni^3+^ and, therefore, enhancing the electrochemical characteristics [21]. Thus, to discriminate the impact of the anti-site disorder on the electrochemical properties of the (1−*x*) LiNi_0.8_Mn_0.1_Co_0.1_O_2_∙*x*Li_2_SO_4_ materials, the samples with different degrees of Li/Ni anti-site disorder were obtained by the variation of the synthesis atmosphere. The powder XRD patterns for the obtained NMC811 samples, prepared from both hydroxide and carbonate precursors and calcined in air (Figure 2a) and in oxygen (Figure 2b) atmosphere, show well-crystallized single-phase α-NaFeO_2_-type layered compounds. All peaks were indexed in the rhombohedral space group *R*-3*m* with the unit cell parameters provided in Table 3.

The lattice parameters, as well as the amount of Ni^2+^/Li^+^ anti-site defects, originated from the partial interchange of the Li^+^ (*r* = 0.76 Å) and Ni^2+^(*r* = 0.69 Å) cations between the 3*b* (i.e., Li) and 3*a* (i.e., Ni) sites [22]. They were calculated from powder XRD patterns for all samples with the Rietveld refinement based on the layered *R*-3*m* structure (Table 3). Small values of the profile reliability factor *R*_p_ and Goodness of fit (GOF) indicated reasonably good refinement results. The observed and calculated powder XRD patterns are shown in Figures S3 and S4 for the air- and oxygen-annealed samples, respectively. As expected, changing the annealing atmosphere from air to oxygen leads to the relative stabilization of the layered structure, as reflected by the declining amount of the Ni^2+^/Li^+^ anti-site defects for the hydroxide and carbonate precursors lithiated in O_2_, compared to ones lithiated in air (Table 3).

**Figure 3:**
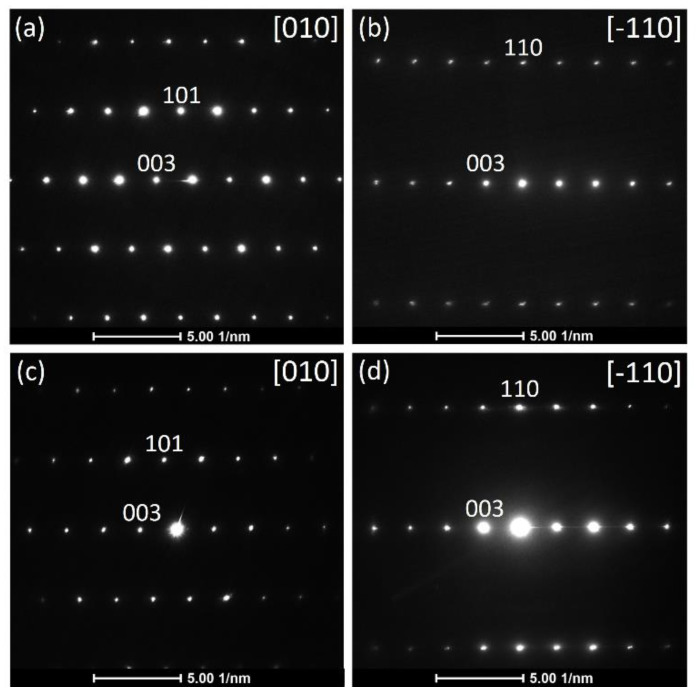
Selected area electron diffraction patterns along [010] and along [-110] zone axes for the HS(o) (**a**,**b**) and HA(o) (**c**,**d**) samples.

Additionally, the crystal structure of Ni-rich NMCs was investigated by selected area electron diffraction (SAED) (Figure 3). All reflections in the SAED patterns of all samples, exemplified with those for the samples HS(o) (Figure 3a,b) and HA(o) (Figure 3c,d), correspond to the layered O3 *R*-3*m* structure. No additional reflections were observed in the SAED patterns, meaning that all investigated samples possessed NMC811 as the only crystalline phase.

**Figure 4:**
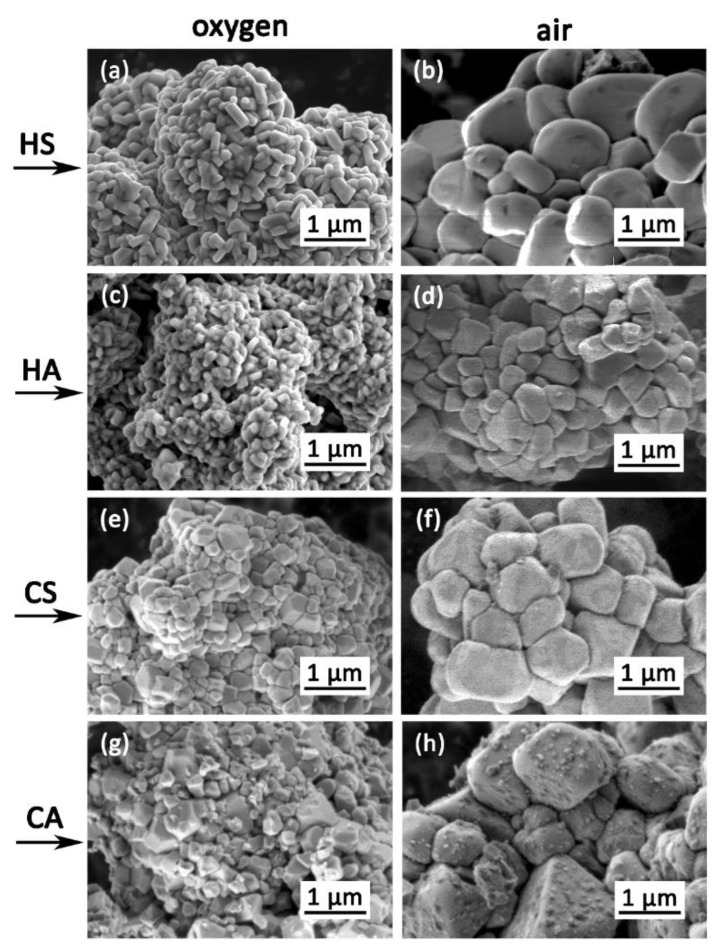
Scanning electron microscopy (SEM) images of primary particles in the NMC811 samples: (**a**)–HS(o), (**b**)–HS(a), (**c**)–CS(o), (**d**)–CS(a), (**e**)–HA(o), (**f**)–HA(a), (**g**)–CA(o), (**h**)–CA(a).

SEM investigated the morphology of the obtained NMC811 cathode materials. The secondary agglomerates in the NMC811 samples retain the secondary agglomerates’ size in their corresponding precursors. However, the precursors’ flake-like primary particles are completely changed into rectangular ones, as shown in Figure 4. The size of the primary particles in the oxygen-annealed samples is clearly smaller (100–500 nm, Figure 4a–d) than the air-annealed particles’ size (0.8–1 µm, Figure 4e–h). Size distributions of the primary particles in the obtained samples are presented in Figure S5.

**Figure 5:**
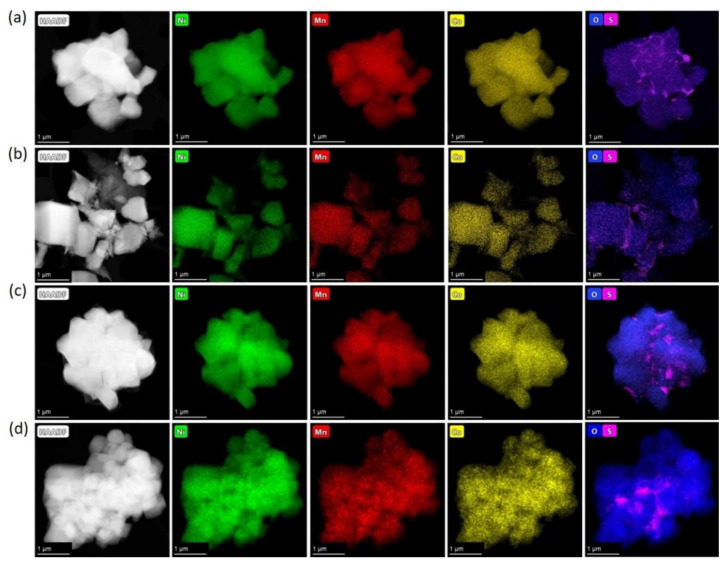
HAADF-STEM images of the (**a**) HS(a); (**b**) CS(a); (**c**) HS(o); (**d**) CS(o) samples with the compositional EDS maps of nickel, manganese, cobalt, oxygen, and sulfur.

**Figure 6:**
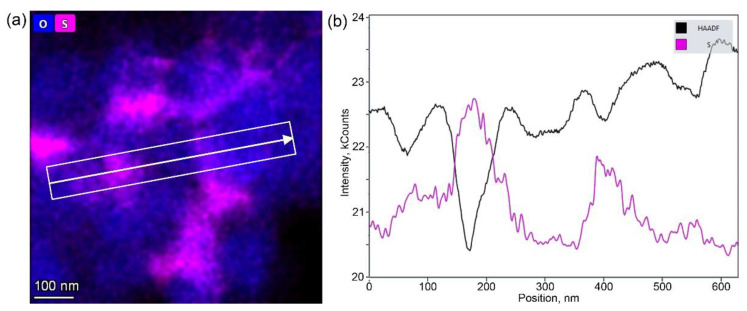
(**a**) Mixed oxygen and sulfur EDS map around several primary grains in the CS(o) sample and (**b**) the profiles of the HAADF (black) and EDS sulfur (pink) signals along the direction marked in the image.

In order to determine the precise elemental composition and distribution of the elements, EDS-STEM analysis was carried out for all NMC811 samples. EDS-STEM elemental maps of nickel, manganese, cobalt, oxygen, and sulfur show that all NMC811 materials, regardless of the synthesis conditions, possess uniform Ni, Mn, and Co distribution (Figure 5). Moreover, both hydroxide and carbonate samples obtained from sulfates include impurities of sulfur-based species. However, powder XRD cannot be detected as any side phase, probably because of the low sulfur content and/or amorphous nature of the sulfur-containing phase, as no other crystalline compounds except NMC811 were observed with the SAED patterns. The atomic percentages of Ni, Mn, Co, and S measured by EDS are listed in Table 4. The cationic composition was in good accordance with the desired NMC811 stoichiometry. Compositional EDS-STEM maps showed that sulfur-containing species were distributed unevenly in the HS(a), CS(a), HS(o), and CS(o) samples (Figure 5a–d), in contrast to very homogeneous sulfur distribution in the co-precipitated precursors (Figure 1). Sulfur appears to be distributed preferentially at the grain boundaries between primary NMC811 crystals and the primary grains’ contact points, as revealed by the correlation between the EDS and HAADF signal profiles in Figure 6 and Figure S6. The sulfur EDS signal was picked at the minima of the HAADF signal corresponding to the intergranular contacts.

**Figure 7:**
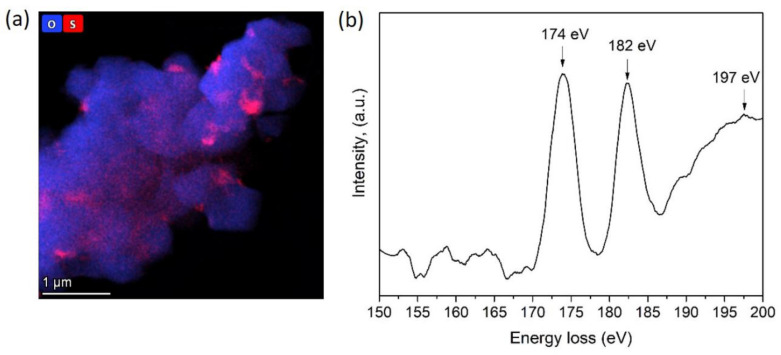
(**a**) Compositional EDS map of oxygen and sulfur and (**b**) S-L_2,3_ EELS spectrum of the HS(o) sample.

The sulfur species’ chemical nature at the grain boundary region of the HS(o) sample was revealed with EELS L_2,3_ sulfur edge (Figure 7). It showed two characteristic sharp pre-peaks at 174 eV, 182 eV, and the broad core edge at 197 eV. According to Reference [23], this type of spectrum corresponds to sulfur in the +6 oxidation state in a tetrahedral oxygen environment, which allows us to conclude that sulfur is presented in the form of sulfate anions. However, EDS did not reveal any enrichment of Ni, Mn or Co cations at the S-rich areas, as well as a presence of any other side cation to counterbalance the negative charge of SO_4_^2−^. Moreover, sulfates of Mn(II), Co(II), and Ni(II) were thermally unstable at the oxidative atmosphere of the calcination step. Li^+^ is the only cation invisible with EDS, which can be bound to the SO_4_^2−^ anion at the given synthesis conditions, particularly considering sufficient thermal stability of Li_2_SO_4_ (melting point of 859 °C, decomposition at >1300 °C). Thus, based on the combination of EDS and EELS results and chemical reasonings, the NMC811 materials, synthesized from TM sulfates through both the hydroxide and carbonate precursors, were considered as composite materials with the general formula (1−*x*) LiNi_0.8_Mn_0.1_Co_0.1_O_2_∙*x*Li_2_SO_4_, where Li_2_SO_4_ is present as an amorphous phase according to XRD and SAED.

**Figure 8:**
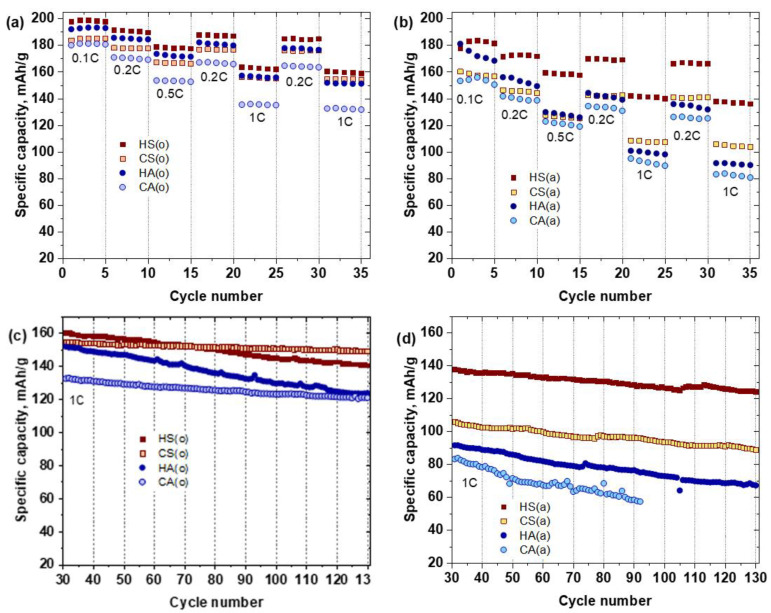
Rate capability at various charge-discharge current densities (**a**,**b**) and cycling performance at 1C rate (**c**,**d**) of the NMC811 samples.

Each sample’s electrochemical properties were evaluated using coin-type lithium half-cells between 2.7–4.3 V vs. Li/Li^+^ at room temperature. Figure 8a,b presents the rate capabilities of the NMC811 samples at different current densities ranging from 20 mA∙g^−1^ to 200 mA∙g^−1^ (0.1C to 1C) and the capacity recovery when the current density returns to 0.2C. The air-annealed samples with the same primary particle size and morphology demonstrated different electrochemical performance. The HS(a) sample exhibits a much higher capacity compared to the HA(a) sample for the whole range of current densities (Table 5). The same tendency was observed for the carbonate-precipitated samples, where the CS(a) sample possessed a higher specific capacity compared to the CA(a) sample (Table 5). The cathode materials precipitated from sulfates always showed higher capacity retention compared to their acetate analogs. Thus, after 130 cycles, HS(a) and CS(a) samples (Figure 7b,d) demonstrated capacity retention of about 91% and 85%, respectively. This was higher than the capacity of the HA(a) and CA(a) samples, which were about 73% and 69%, respectively. The initial discharge capacity was markedly enhanced by switching the calcination atmosphere from air to oxygen due to suppressing the Ni^2+^/Li^+^ anti-site disorder, as revealed with the Rietveld refinement (Table 3). Given Li diffusion limitations, the HS(o) and HA(o) samples with primary particle size about 100–300 nm demonstrated higher discharge capacity of ~199 and 193 mA∙g^−1^, respectively, compared to 186 and 181 mA∙g^−1^ for the CS(o) and CA(o) samples with primary particle size of about 0.5–1 µm (Table 5). Simultaneously, the initial discharge capacity of the oxygen-annealed sulfate samples is always higher than that of the oxygen-annealed acetate samples. The capacity retention and rate capability for the sulfate/acetate pairs were also different. The HS(o) sample demonstrated higher capacity retention of 91%, compared to that of HA(o) (81%) (Table 5). The same tendency was observed for the samples obtained from the carbonate precursor: the CS cathode materials possessed enlarged stability at different current densities (0.1C, 0.2C, 0.5C, and 1C) and prolonged cycling. Moreover, the rate capability of carbonate-made cathode material precipitated from sulfates was greater than that of the sample obtained from acetates, giving at least 10–15% growth in discharge capacity at elevated current densities. In addition, the comparison to the analogous NMC811 samples obtained in Reference [15] using the same synthesis conditions showed that sulfur-containing samples prepared in the present work demonstrated improved electrochemical properties.

The sulfate-containing samples, obtained in both atmospheres, as well as the acetate-made samples, annealed in oxygen, recovered their original discharge capacity when the current density returned to 0.2C. This result demonstrated that the capacity fade at high current densities was caused by diffusion limitations rather than irreversible structural changes [24] (Figure 7a,b).

**Figure 9:**
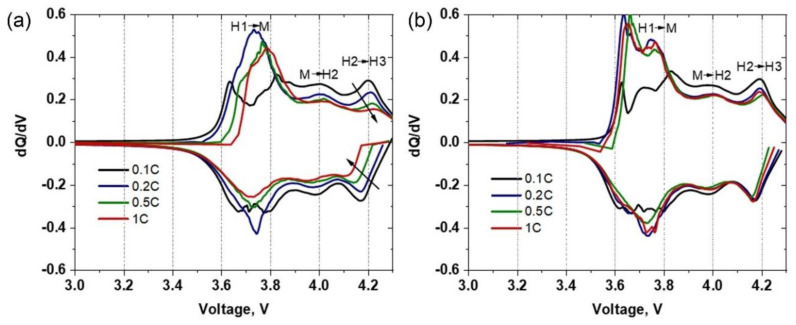
Differential capacity vs. voltage profiles of (**a**) HS(o) and (**b**) HA(o) samples taken at different C-rates.

The differential capacity (*d*Q/*d*V) plots for sulfur-containing HS(o) composite and single-phase NMC811 HA(o) samples are presented in Figure 9. The most important features, which could be observed from differential capacity curves for Ni-rich NMCs, are the phase transformations, appearing throughout the electrochemical cycling. Here, the *d*Q/*d*V profiles of HS(o) and HA(o) samples show three peaks in the anodic branch between 3.5 V and 4.3 V, which are related to three phase transitions: hexagonal-to-monoclinic (H1→M), monoclinic-to-hexagonal (M→H2), and hexagonal-to-hexagonal (H2→H3) (Figure 9a,b) [25]. The corresponding reduction peaks are presented in the cathodic branch. It is generally believed that the H2 → H3 phase transition is associated with structural transformation through transition-metal ion migration to the lithium layer, accompanied by a significant decrease of the *c* lattice parameter. This effect leads to the formation of microcracks in secondary particles, thereby promoting the material’s degradation [8,9]. Besides, at a highly charged state, TM ions’ active migration to the Li sites leads to irreversible crystal structure changes, blocking of Li-ions migration channels, and following drop of Li diffusivity, resulting in a decrease of the electrochemical performance of the cathode materials [26].

As reported previously [27,28], sharp oxidation peaks in the *d*Q/*d*V plots of Ni-rich NMC cathode materials usually represent abrupt and possibly detrimental structural changes, while broader peaks with low intensity indicate protracted phase transitions. The *d*Q/*d*V curves, plotted at 0.1C, 0.2C, 0.5C, and 1C current densities, show that the presence of a sulfur-containing compound in the HS(o) sample reduces the abruptness of the phase transitions at different current densities. This is because the intensities of redox peaks in the *d*Q/*d*V plot of the HS(o) cathode (Figure 9a) show a more rapid decline with an increase of current density compared to those of HA(o) (Figure 9b). The most noticeable suppression of the redox peaks is observed for the H2→H3 phase transition, which is believed to be responsible for the capacity loss of Ni-rich NMCs [29]. Thus, the presence of sulfur-containing species helps to suppress the mentioned deleterious phase transition [7].

The half-cells with HS(o)- and HA(o)-based electrodes after 130 cycles were disassembled inside a glovebox, and the electrodes were investigated by powder XRD analysis. The powder XRD patterns can still be fitted using the Rietveld refinement with the rhombohedral space group *R*-3*m* (Figure S7). As for pristine cathode materials, the amount of Ni^2+^/Li^+^ anti-site defects were calculated from powder XRD patterns for both cycled HS(o)- and HA(o)-based electrodes. It was found that the amount of anti-site defects in HA(o) active cathode material increased significantly from 2.12 to 13.96% after cycling, while in HS(o), it barely increased from 2.54 to 4.12% (Table S1).

**Figure 10:**
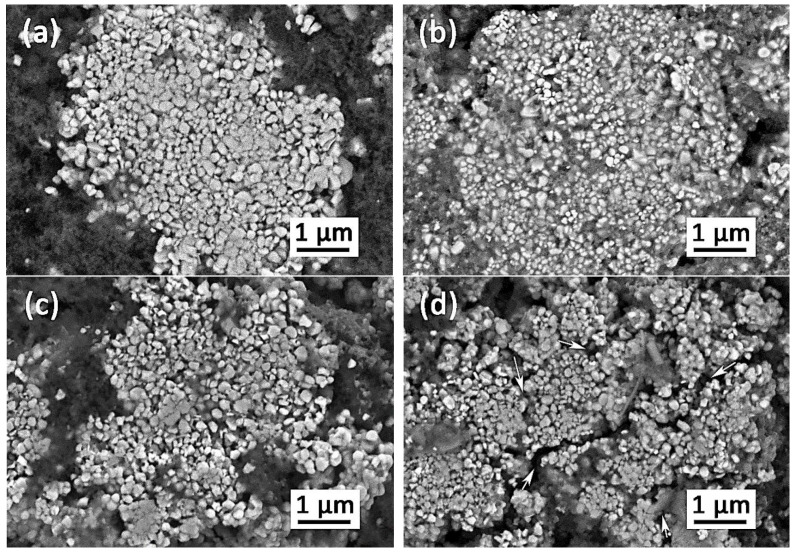
Scanning electron microscopy images for the pristine HS(o) (**a**) and HA(o) (**c**) electrodes and the corresponding electrodes after 130 charge/discharge cycles (**b**,**d**). The cracks in the secondary agglomerates of the cycled HA(o) electrode are marked with arrows.

Given the high voltage H2→H3 phase transition associated with irreversible mechanical degradation of the material, i.e., the formation of microcracks within the secondary particles and detachment of cathode particles from cathode slurry binder, a study of cathode morphology was carried out via comparative SEM of the as-prepared HS(o)- and HA(o)-based electrodes and the corresponding electrodes after 130 charge/discharge cycles (Figure 10). While the HS(o) electrode retains its integrity after cycling (Figure 10a,b), large cracks across the cathode secondary particles as well as numerous detachments between the secondary particles and binder were observed in the HA(o)-based cathode (Figure 10c,d). These detrimental issues in the HA(o)-based cathode are mainly associated with mechanical stresses induced by repetitive volume expansion and contraction of the primary particles during electrochemical cycling [30]. Still, no statistically relevant changes in primary particle size were detected from the cycled cathodes’ SEM images compared to the pristine ones (Figure S8).

**Figure 11:**
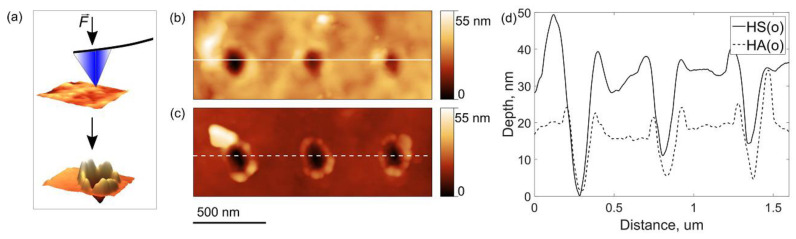
The scheme of the AFM nanoindentation experiment (**a**), AFM topography images of typical indents on (**b)** HS(o) and (**c**) HA(o) secondary particles and (**d**) corresponding cross-section depth profiles.

The improved mechanical behavior of the HS(o)-based cathode could be attributed to the fact that amorphous Li_2_SO_4_ serves as a soft binder linking harder NMC811 primary particles into a composite. Given the amorphous nature of Li_2_SO_4_, it may help to relieve stresses that emerged during cycling and, therefore, preserve the cathode integrity [31]. This conjecture has been tested by AFM nanoindentation experiments (Figure 11a). Figure 11b,c illustrates typical indents obtained on the secondary particles in the HS(o) and HA(o) samples at 130 µN applied force. The corresponding indentation curves are shown in Figure S9. From the profiles in Figure 11d, one can see that the indents are deeper in the HS(o) sample. Simultaneously, the expelled material around the indents is not fractured on the HS(o) sample and strongly fractured on the HA(o) sample. Crack formation in the HA(o) sample becomes evident from the load-displacement curves (Figure S7), demonstrating pop-in events caused by a sudden increase of the indenter penetration due to cracking [32]. It points out that the amorphous Li_2_SO_4_ species at the boundaries between the primary particles and their intergranular contacts accommodate part of the mechanical stress and hold the primary particles together, thereby effectively preventing the secondary particles’ mechanical fracture. In light of the nanoindentation experiments, the (1−*x*) LiNi_0.8_Mn_0.1_Co_0.1_O_2_∙*x*Li_2_SO_4_ materials behave as composites consisting of primary particles of the NMC811 phase with higher hardness linked together with softer Li_2_SO_4_ binder. It is well known that in such composites decreasing hardness is accompanied by increasing interfacial fracture toughness [33,34]. The quantitative estimates of the Young’s modulus *E* and indentation hardness *H* values for the HS(o) (*E* = 165(80) GPa, *H* = 2.53(60) GPa) and HA(o) (*E* = 200(30) GPa, *H* = 3.35(40) GPa) samples strongly support this conjecture.

## 4. Discussion

We conducted a comparative investigation of the NMC811 samples obtained through the coprecipitation method from different TM sources using either carbonate or hydroxide routes and lithiation at different annealing atmospheres. The investigation allowed us to discriminate the role in electrochemical behavior of minor sulfur impurity from the influence of other important factors, such as crystal structure, homogeneity of the TM distribution, amount of anti-site defects, size of the primary particles, and secondary agglomerates. Sulfur, which is present as amorphous Li_2_SO_4_ at the grain boundaries and intergranular contacts, remarkably enhances capacity retention over prolonged electrochemical cycling, improves rate capability and initial discharge capacity of the NMC811 cathodes.

The attractive point is that grain boundary modification with Li_2_SO_4_ does not require any additional synthesis step and occurs spontaneously if the hydroxide or carbonate precursors are precipitated from TM sulfates’ solutions. The solutions are the most common initial ingredients for the production of the NMC-based cathode materials. Sulfur is distributed homogeneously over the precursor’s particles without any sign of segregation, and numerous attempts to eradicate it by repetitive rinsing with water were unsuccessful. Taking into account the absence of other crystalline phases in the precursors, one might suggest that sulfur enters as SO_4_^2−^ into the crystal structure of the precursors forming the solid solutions M(OH)_2__−2*x*_(SO_4_)*_x_* or M(CO_3_)_1__−*x*_(SO_4_)*_x_*∙H_2_O (M—transition metals), where the substitution range does not exceed few percent. It opens up the interesting possibility to vary sulfur content by changing the TM sulfate solution’s concentration, the sulfate injection rate, and precipitating and chelating agents into the reactor volume and controlling pH. Therefore, it gives extra dimensions to the already matured coprecipitation synthesis technique. The high-temperature lithiation step leads to segregation of SO_4_^2−^ anions at the boundaries between the primary NMC811 crystallites indicating negligible solubility of sulfate in the layered structure of the NMC811 complex oxide. Indeed, it is in line with the low thermal stability of the Ni, Mn, and Co sulfates in air or in oxygen, whereas Li_2_SO_4_ remains the only stable sulfate in the system. Surprisingly, neither XRD nor SAED detects crystalline Li_2_SO_4,_ strongly suggesting its amorphous nature despite the relatively high temperature of the lithiation step of 750–850 °C. The exact structure of Li_2_SO_4_ and its dependence on the processing conditions calls for further in-depth studies.

The mechanism through which Li_2_SO_4_ affects the electrochemical behavior is multimodal. Interestingly, lithium sulfate is a good Li-ion conductor in its high-temperature α-phase above 575 °C, after undergoing a first-order phase transition. However, at lower temperatures, crystalline Li_2_SO_4_ demonstrates relatively poor Li-ion conductivity [35]. However, amorphous Li_2_SO_4_ behaves differently, as demonstrated with improved Li-ion transport in the mixtures of the layered, complex oxide cathodes and amorphous Li_2_SO_4_ employed in all-solid-state Li-ion batteries [17,36]. Unlike previous research [17,36], in the present work, improving Li-ion transport in the NMC811 secondary agglomerates by enhancing the intergranular contacts with amorphous Li_2_SO_4_ layers positively impacted the rate capability of the sulfur-containing cathodes. We achieved this without noticeable reduction of energy density because of a much smaller amount of Li_2_SO_4_. Simultaneously, grain boundary modification smoothens the phase transitions driving the material towards more solid-solution-like behavior that should also increase the rate capability. A very significant difference is observed in the amount of accumulated Ni^2+^/Li^+^ anti-site disorder in the cathodes with and without sulfur. Three times lower anti-site disorder in the sulfur-containing HS(o) cathode is coupled with the partial suppression of the H2→H3 phase transition, which is associated with oxygen loss at high potentials through the reaction with the electrolyte driving the layered structure towards the rock-salt one [7,37,38,39]. Thus, the amorphous Li_2_SO_4_ layer at the grain boundaries can partially protect the surface of the primary NMC811 crystallites from unwanted reaction with the electrolyte at elevated potentials. Finally, the composite (1−*x*) LiNi_0.8_Mn_0.1_Co_0.1_O_2_∙*x*Li_2_SO_4_ cathode preserves its mechanical integrity after prolonged cycling, unlike its single-phase NMC811 counterparts. Amorphous Li_2_SO_4_ acts as a soft binder linking the harder NMC811 primary particles, thereby lowering the hardness of the secondary agglomerates and at the same time improving the interfacial fracture toughness making the agglomerates more resistant to cracking associated with interrupting the charge transfer pathways and compromising the mechanical stability of the cathode [9].

## 5. Conclusions

(1−*x*) LiNi_0.8_Mn_0.1_Co_0.1_O_2_∙*x*Li_2_SO_4_ (*x* < 0.005) composite cathode materials for Li-ion batteries were prepared through either hydroxide or carbonate coprecipitation techniques followed by high-temperature lithiation. Amorphous Li_2_SO_4_, located at the grain boundaries and intergranular contacts of the primary NMC811 crystallites, enhances the capacity retention and rate capability of the cathodes cycled within the 2.7–4.3 V vs. Li/Li^+^ voltage window. The positive effect is associated with suppressing the high voltage phase transition and accumulated anti-site disorder after prolonged cycling and improving mechanical integrity of the secondary agglomerates by increasing interfacial fracture toughness by linking primary NMC811 particles with soft Li_2_SO_4_ binder. The present work highlights a possible route for further improvement of Ni-rich NMCs’ electrochemical properties through dedicated engineering of the grain boundaries’ structure and composition.

## Figures and Tables

**Table 1:** Synthesis conditions of the NMC811 precursors.

Sample	Source of TM	C_TM_, M	Precipitant Concentration, M	pH
HS	M^2+^SO_4_	2	4.3 M NaOH	11.5
HA	M^2+^(CH_3_COO)_2_	0.5	1M NaOH	11.5
CS	M^2+^SO_4_	2	2 M Na_2_CO_3_	7.8
CA	M^2+^(CH_3_COO)_2_	0.5	0.5 M Na_2_CO_3_	7.8

**Table 2:** Quantitative elemental analysis for the HS, HA, CS, and CA precursors.

Precursor	Ni, mol. Fraction	Mn, mol. Fraction	Co, mol. Fraction	S, mol.% of TM
HS	0.819 ± 0.016	0.086 ± 0.011	0.095 ± 0.005	1.221 ± 0.158
HA	0.833 ± 0.049	0.078 ± 0.036	0.088 ± 0.014	-
CS	0.802 ± 0.003	0.085 ± 0.003	0.099 ± 0.001	1.391 ± 0.122
CA	0.796 ± 0.055	0.102 ± 0.053	0.102 ± 0.005	-

**Table 3:** Rietveld refinement results for the NMC811 samples.

Sample	*a*, Å	*c*, Å	V, Å^3^	Ni^2+^ in Li Site, %	R_p_, %	GOF
HS(a)	2.8792(6)	14.2308(1)	101.573(3)	4.63	2.24	1.90
HA(a)	2.8748(1)	14.2131(8)	101.729(2)	5.95	2.09	1.39
CS(a)	2.8776(4)	14.2213(3)	101.970(3)	6.34	2.13	1.38
CA(a)	2.8759(3)	14.2165(3)	101.830(3)	6.92	2.43	1.47
HS(o)	2.87212(2)	14.2044(2)	101.4750(2)	2.54	1.92	1.43
HA(o)	2.87010(2)	14.1989(2)	101.2928(2)	2.12	1.92	1.30
CS(o)	2.87227(2)	14.1951(2)	101.4191(1)	3.05	2.73	1.95
CA(o)	2.87345(2)	14.1881(2)	101.4525(2)	4.43	3.03	2.22

**Table 4:** Quantitative elemental analysis for NMC811 samples.

Sample	Ni, mol. Fraction	Mn, mol. Fraction	Co, mol. Fraction	S, mol.% from TM
HS(a)	0.822 ± 0.035	0.095 ± 0.026	0.099 ± 0.006	0.43 ± 0.24
HA(a)	0.786 ± 0.005	0.110 ± 0.003	0.121 ± 0.005	-
CS(a)	0.783 ± 0.010	0.097 ± 0.002	0.106 ± 0.002	0.25 ± 0.17
CA(a)	0.788 ± 0.015	0.099 ± 0.005	0.103 ± 0.004	-
HS(o)	0.792 ± 0.037	0.103 ± 0.025	0.099 ± 0.015	0.49 ± 0.35
HA(o)	0.781 ± 0.018	0.110 ± 0.013	0.109 ± 0.006	-
CS(o)	0.792 ± 0.009	0.099 ± 0.004	0.106 ± 0.006	0.27 ± 0.21
CA(o)	0.785 ± 0.016	0.107 ± 0.016	0.108 ± 0.002	-

**Table 5:** Electrochemical performance parameters for the NMC811 samples.

Sample	Discharge Capacity				Capacity Retention after 100 Cycles at 1C, %
	0.1C, mA h g^−1^	0.2C, mA h g^−1^	0.5C, mA h g^−1^	1C, mA h g^−1^	
HS(a)	184	173	160	142	91
HA(a)	181	156	129	101	73
CS(a)	160	147	128	109	85
CA(a)	156	142	123	95	69 ^1^
HS(o)	199	192	179	164	90
HA(o)	193	186	174	157	81
CS(o)	186	178	168	156	97
CA(o)	181	171	154	135	91
NMC811 [15]	196	180	160	150	86

^1^-measured after 93 cycles.

## Supplementary Materials

The following are available online at https://www.mdpi.com/2079-4991/10/12/2381/s1, Figure S1: Powder XRD patterns of (a) hydroxide precursor with β-Ni(OH)2 structure (ICDD #74-2075), precipitated from sulfates (HS) and (b) carbonate precursor, isostructural to NiCO3∙H2O (ICDD #12-0276), precipitated from sulfates (CS), Figure S2: Scanning electron microscopy images of different magnification for the obtained precursors in hydroxide (a,b) HS, (c,d) HA and carbonate (e,f) CS, (g,h) CA forms, Figure S3: Powder XRD patterns and Rietveld refinement profiles of (a) HS(a); (b) HA(a); (c) CS(a); (d) CA(a), Figure S4: Powder XRD patterns and Rietveld refinement profiles of (a) HS(o); (b) HA(o); (c) CS(o); (d) CA(o), Figure S5: Primary particles size distributions in the NMC811 samples: (a) HS(o), (b) HS(a), (c) CS(o), (d) CS(a), (e) HA(o), (f) HA(a), (g) CA(o), (h) CA(a), Figure S6: (a) Mixed oxygen and sulfur EDS map around several primary grains in the CS(o) sample and (b) the profiles of the HAADF (black) and EDS sulfur (pink), and oxygen (blue) signals along the direction marked in the image, Figure S7: Powder XRD patterns and Rietveld refinement profiles of the (a) HS(o)- and (b) HA(o)-based cathodes after 130 charge/discharge cycles (reflections from the Al foil current collector are indicated with arrows), Figure S8: Primary particles size distributions in the pristine HS(o) (a) and HA(o) (c) electrodes and the corresponding electrodes after 130 charge/discharge cycles (b,d), Figure S9: Typical nanoindentation curves measured on the secondary particles in the HS(o)- and HA(o)-based cathodes. Crack-induced pop-in event is marked with an arrow, Table S1: Rietveld refinement results for the NMC811 samples after 130 charge/discharge cycles.
