# Supplementary material for: Sulfate-Containing Composite Based on Ni-Rich Layered Oxide LiNi0.8Mn0.1Co0.1O2 as High-Performance Cathode Material for Li-ion Batteries
Source: Nanomaterials (Basel). 2020 Nov 29;10(12):2381. doi: 10.3390/nano10122381 (PMC7759786; doi:10.3390/nano10122381)
Supplement: Supplementary file 1 [file nanomaterials-10-02381-s001.pdf]

Supplementary materials

Sulfate-containing composite based on Ni-rich layered oxide $LiNi_{0.8}Mn_{0.1}Co_{0.1}O_2$ as high-performance cathode material for Li-ion batteries.

Aleksandra A. Savina[1], Elena D. Orlova[1], Anatolii V. Morozov[1], Sergey Yu. Luchkin[1], Artem M. Abakumov[1]

[1] Center for Energy Science and Technology, Skolkovo Institute of Science and Technology, Bolshoy Boulevard 30, bld. 1, 121205 Moscow, Russia.

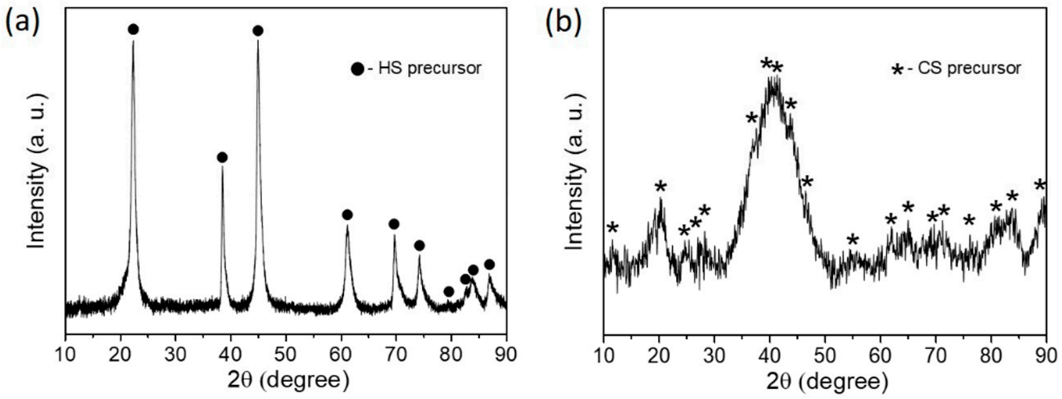

Figure S1. Powder XRD patterns of (a) hydroxide precursor with β-$Ni(OH)_2$ structure (ICDD #74-2075), precipitated from sulfates (HS) and (b) carbonate precursor, isostructural to $NiCO_3 \cdot H_2O$ (ICDD #12-0276), precipitated from sulfates (CS).

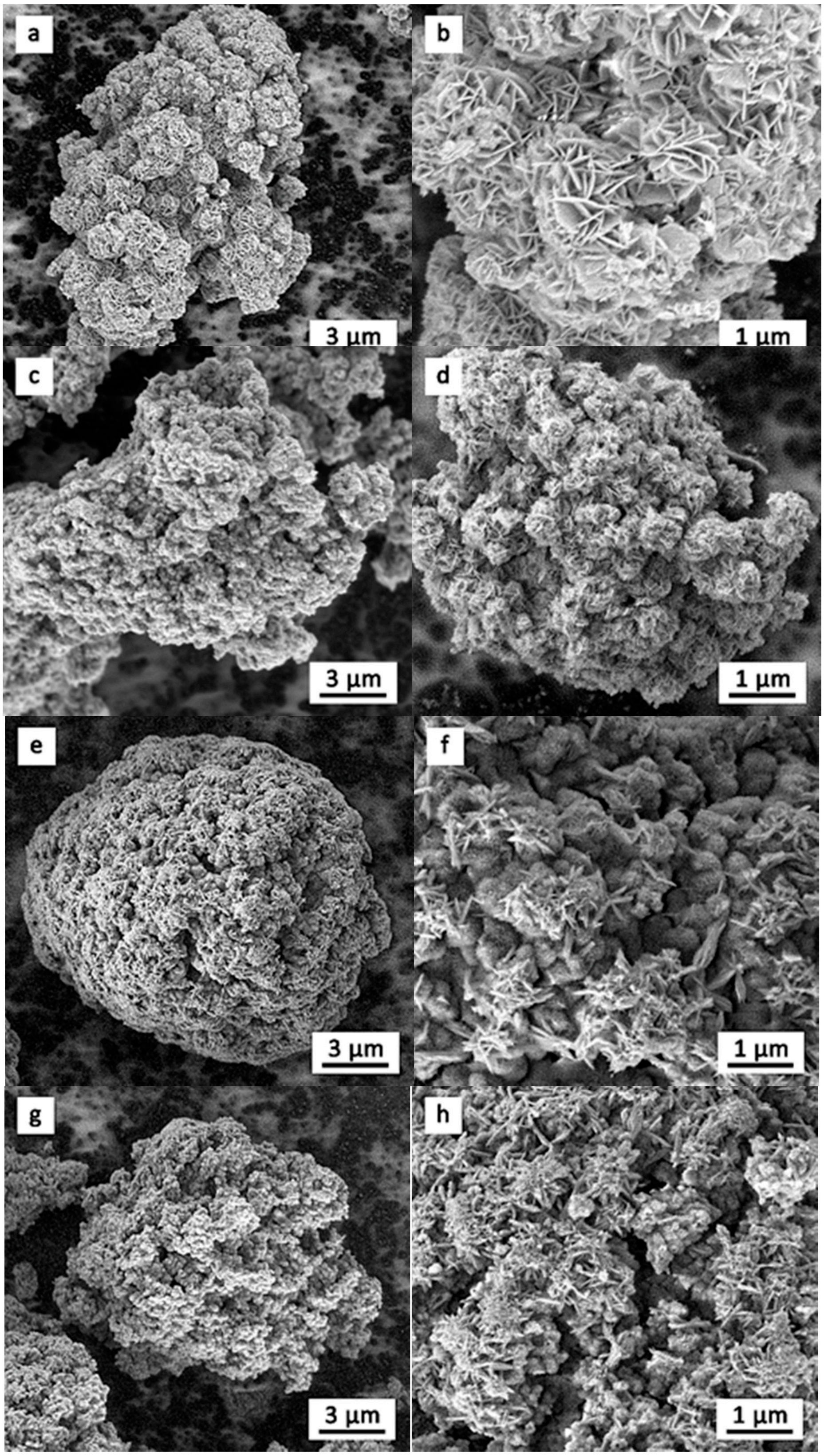

Figure S2. Scanning electron microscopy images of different magnification for the obtained precursors in hydroxide (a, b) HS, (c, d) HA and carbonate (e, f) CS, (g, h) CA forms.

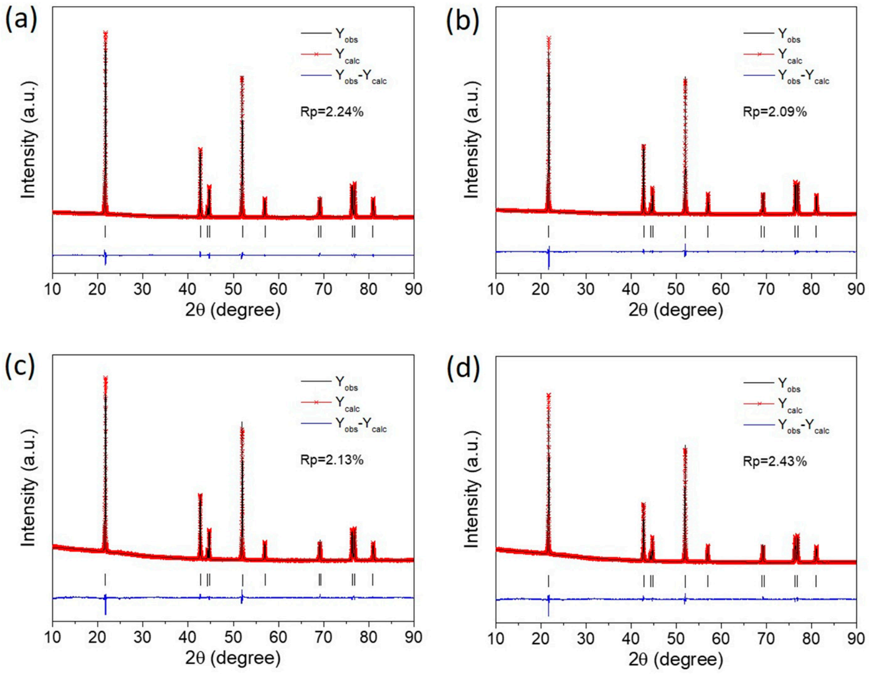

Figure S3. Powder XRD patterns and Rietveld refinement profiles of (a) HS(a); (b) HA(a); (c) CS(a); (d) CA(a).

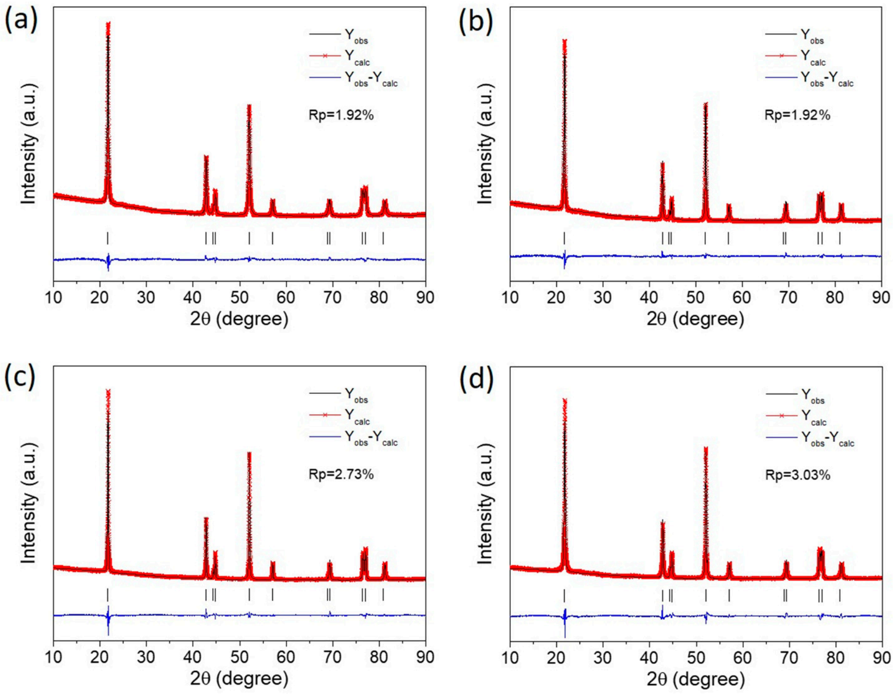

Figure S4. Powder XRD patterns and Rietveld refinement profiles of (a) HS(o); (b) HA(o); (c) CS(o); (d) CA(o).

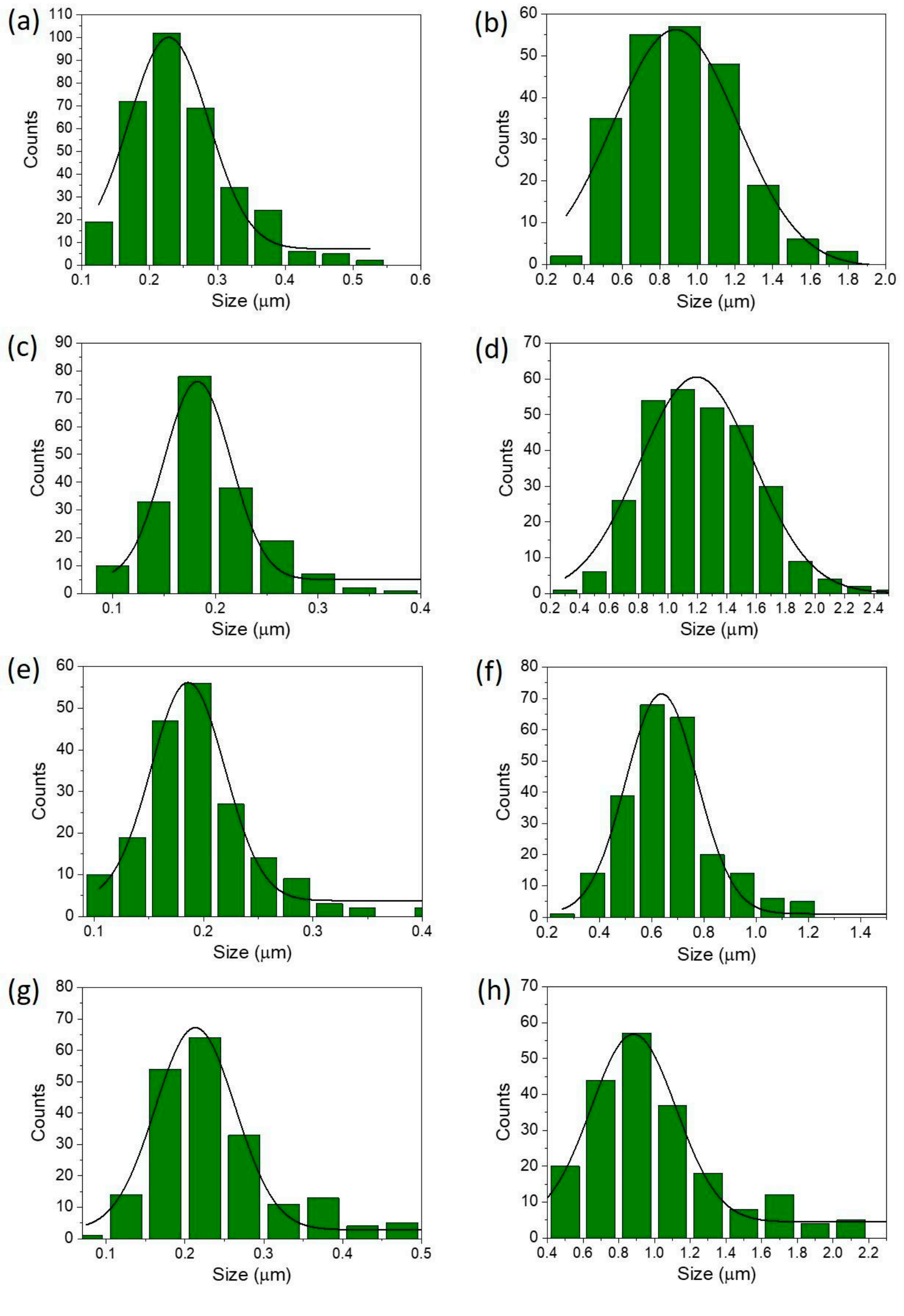

Figure S5. Primary particles size distributions in the NMC811 samples: (a) - HS(o), (b) - HS(a), (c) - CS(o), (d) - CS(a), (e) - HA(o), (f) - HA(a), (g) - CA(o), (h) - CA(a).

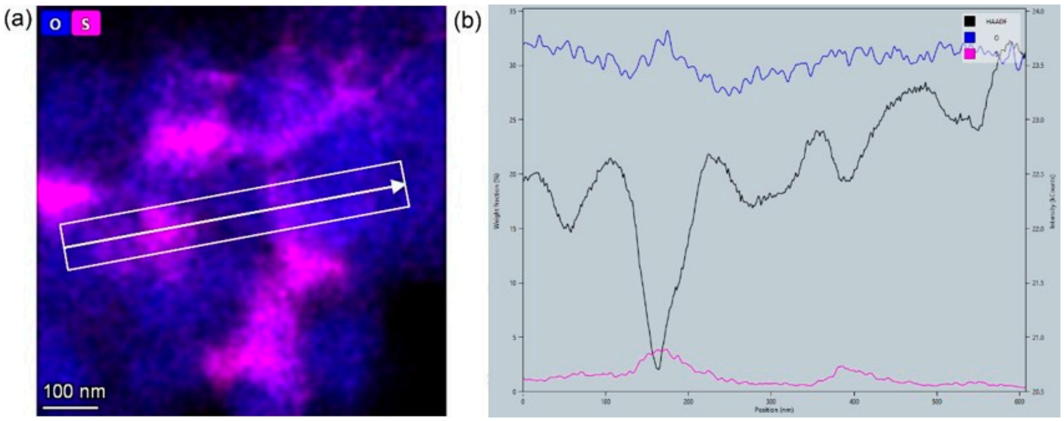

Figure S6. (a) Mixed oxygen and sulfur EDS map around several primary grains in the CS(o) sample and (b) the profiles of the HAADF (black) and EDS sulfur (pink), and oxygen (blue) signals along the direction marked in the image.

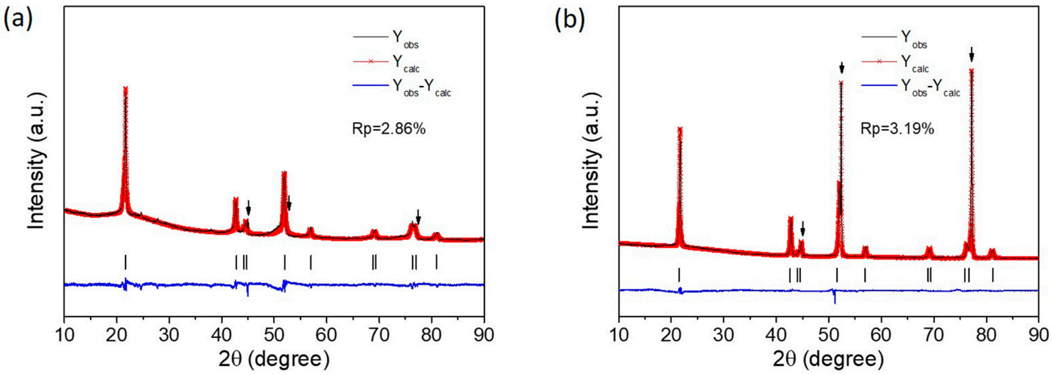

Figure S7. Powder XRD patterns and Rietveld refinement profiles of the (a) HS(o)- and (b) HA(o)- based cathodes after 130 charge/discharge cycles (reflections from the Al foil current collector are indicated with arrows).

Table S1. Rietveld refinement results for the NMC811 samples after 130 charge/discharge cycles.

**Sample**	***a*, Å**	***c*, Å**	**V, Å^3**	**Ni^{2+} in Li site, %**	**Rp, %**
HS(o) cycled	2.88103(5)	14.2402(3)	102.362(3)	4.12	2.86
HA(o) cycled	2.87519(4)	14.2477(4)	102.002(3)	13.96	3.19

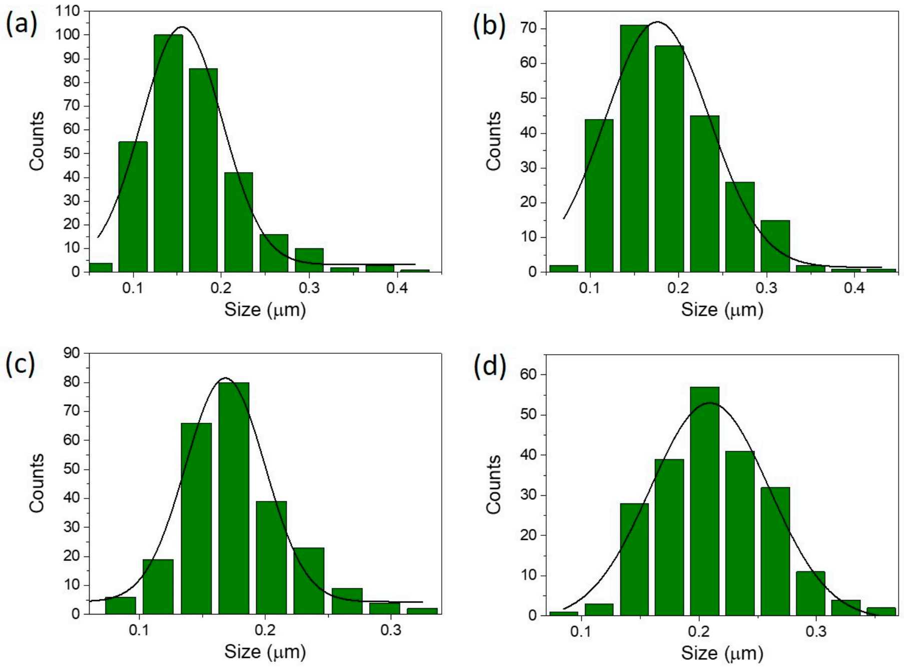

Figure S8. Primary particles size distributions in the pristine HS(o) (a) and HA(o) (c) electrodes and the corresponding electrodes after 130 charge/discharge cycles (b, d).

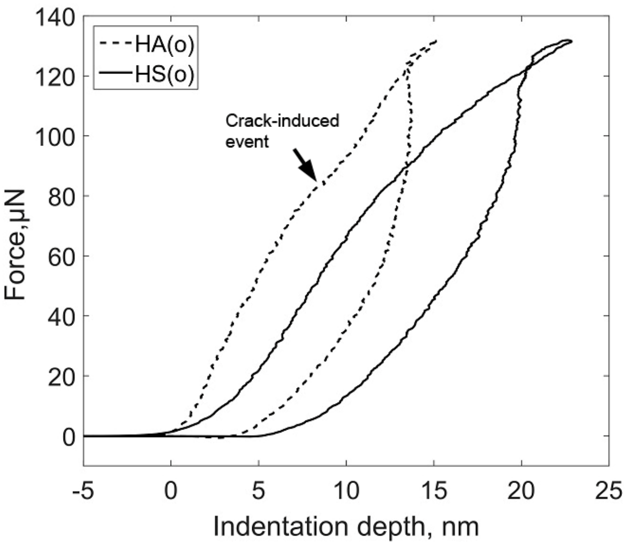

Figure S9. Typical nanoindentation curves measured on the secondary particles in the HS(o)- and HA(o)- based cathodes. Crack-induced pop-in event is marked with an arrow.
